# A Qualitative Study of the Impact of COVID-19 on Smoking Behavior for Participants in a Post-Hospitalization Smoking Cessation Trial

**DOI:** 10.3390/ijerph18105404

**Published:** 2021-05-19

**Authors:** Kristina Schnitzer, Sarah Jones, Jennifer H. K. Kelley, Hilary A. Tindle, Nancy A. Rigotti, Gina R. Kruse

**Affiliations:** 1 Tobacco Research and Treatment Center, Massachusetts General Hospital, Boston, MA 02114, USA; KSCHNITZER@PARTNERS.ORG (K.S.); SJONES55@mgh.harvard.edu (S.J.); JKELLEY4@mgh.harvard.edu (J.H.K.K.); nrigotti@partners.org (N.A.R.); 2 Department of Psychiatry, Massachusetts General Hospital, Boston, MA 02114, USA; 3 Harvard Medical School, Boston, MA 02115, USA; 4 Health Policy Research Center, Mongan Institute, Massachusetts General Hospital, Boston, MA 02114, USA; 5 Vanderbilt University Medical Center, Nashville, TN 37232, USA; hilary.tindle@vumc.org; 6 Geriatric Research Education and Clinical Centers (GRECC), Veterans Affairs Tennessee Valley Healthcare System, Nashville, TN 37212, USA; 7 Division of General Internal Medicine, Department of Medicine, Massachusetts General Hospital, Boston, MA 02114, USA

**Keywords:** COVID-19, smoking cessation, qualitative interviews, Transactional Model of Stress

## Abstract

(1) Background: COVID-19 has substantially altered individual environments and behaviors. We aim to explore the impact of COVID-19 on the smoking behavior of individuals trying to quit tobacco. (2) Methods: This study presents a qualitative analysis of individual interviews focused on perceived impacts of the COVID-19 pandemic on tobacco use among 39 participants in the Helping HAND 4 (HH4) post-hospitalization smoking cessation trial (NCT03603496). (3) Results: Emergent impacts of COVID-19 included change in routine, isolation, employment changes, and financial challenges; these in turn were associated with boredom, altered cravings and triggers, and increased stress. The availability of effective coping mechanisms instead of smoking to deal with stress heavily influenced subsequent smoking behavior. These results were triangulated with the Transactional Model of Stress, providing a framework to elucidate connections between factors such as perceived control, self-efficacy, and dispositional coping style, and highlighting potential areas for intervention. (4) Conclusions: Results suggest that stress during the COVID-19 pandemic may undermine effective coping skills among individuals enrolled in a post-hospitalization smoking cessation trial. Strengthening effective coping skills (e.g., minimizing the use of tobacco as a default stress response) and increasing perceived control and self-efficacy are promising intervention targets.

## 1. Introduction

The Coronavirus Disease 2019 (COVID-19) has resulted in unprecedented physical, mental, economic, and societal disruption on a global scale. Similar to other man-made and natural disasters, the COVID-19 pandemic has brought elevated stress, disruption of routines, and financial burden that may increase smoking. Existing data have demonstrated an increase in nicotine dependence during disasters such as Hurricane Katrina, 9/11 and the Oklahoma City bombing [1,2,3,4,5]. In the case of 9/11, the effect was sustained with increased smoking observed for six months after the event [6]. The effects of population-level financial events, like economic recession, may also impact smoking although the effect is less clear. In the 2008 recession, UK smokers smoked more in the setting of personal financial strain while adults in Iceland smoked less [7,8]. Possible drivers of changes in smoking in the setting of these disasters include increases in stress, depression and anxiety.

Specific characteristics of the COVID-19 pandemic may also amplify its impact on smokers. First, there may be greater feelings of isolation due to social distancing. Second, smokers may feel enhanced vulnerability stemming from the respiratory manifestations of the virus. Alternatively, the combination of disrupted routines and enhanced vulnerability may encourage cessation, in a manner resembling a hospitalization, where perceptions of smoking risks and quitting benefits can combine to promote cessation [9]. At this time, there is not robust literature describing the impacts of the pandemic on smoking behaviors. Exploratory, hypothesis-generating methods are urgently needed to understand the factors influencing tobacco behaviors during the pandemic if we are to mitigate the risks of ongoing tobacco use.

This study aimed to examine, through a qualitative lens, individuals’ experiences of COVID-19 and resultant stress on smoking behaviors among participants in a post-hospitalization smoking cessation trial and subsequently to place these findings within a broader context of existing theories of health behavior [10].

## 2. Materials and Methods

This cross-sectional study is a qualitative analysis of audio-recorded telephone interviews conducted with participants of the HH4 trial. HH4 (NCT03603496) is a two-arm randomized clinical trial conducted across three sites (Massachusetts General Hospital [MGH], University of Pittsburgh Medical Center [UPMC], and Vanderbilt University Medical Center [VUMC], USA). It tested the effectiveness of Personalized Tobacco Care Management (PTCM) which provided nicotine replacement therapy (NRT) and smoking cessation support from a certified tobacco treatment specialist (CTTS), versus eReferral to a state Quitline which typically includes free counseling and smoking cessation medication. The primary outcome was validated tobacco abstinence six months post-hospital discharge. The full trial protocol is available [11]. Ethical approval was obtained from the IRBs of all participating institutions, including supplemental approval for the qualitative component represented here.

### 2.1. Parent Study Participants

The parent HH4 trial enrolled 1416 individuals; eligibility criteria included all adult daily tobacco users admitted to one of the three study hospitals (MGH, UPMC, VUMC) from September 2018 to March 2020 who were willing to try to quit smoking after discharge and accept a prescription for NRT.

### 2.2. Baseline Survey Measures

A baseline survey during the initial hospital stay assessed the participants’ sociodemographic characteristics and smoking history. Additional surveys at baseline included the Generalized Anxiety Disorder 7-item scale (GAD-7) [12], the Patient Health Questionnaire 8-item depression scale (PHQ-8) [13], the Life Orientation Test (LOT-R) [14], and the Brief Resilience Scale (BRS) [15]. Planned study surveys assessed outcomes at 1, 3, and 6-months post-enrollment.

### 2.3. COVID Qualitative Interview

A subset of trial participants was invited to participate in a qualitative telephone interview. Study participants from all three study sites who were scheduled to complete either a 3 or 6-month follow-up survey by telephone from 13 April 2020 (date of supplemental IRB approval) through 30 October 2020 (date of last 6-month survey collection) were invited to participate in a supplemental qualitative interview. We chose telephone rather than videoconferencing as the interview mode to align with other study activities and to allow participants without internet-capable devices to participate. This approach was intended to maximize the variation in experiences during the pandemic including potential geographic impacts as well as differences in quit behaviors at different time points relative to study enrollment. The interview aimed to understand individuals’ experiences with COVID-19 and pandemic impacts on their tobacco use. Thirty-nine individuals agreed to participate in the supplemental COVID-focused, one-on-one, audio-recorded qualitative interview (MGH n = 15, UPMC n = 15, VUMC n = 9). Interviewees provided verbal informed consent for their recorded calls to be analyzed. Four of these individuals, who had both a 3- and 6-month follow-up during the aforementioned time period, completed the qualitative interview twice. Individuals received $20 for their participation in the qualitative interview. The remuneration amount for this activity was selected to align with other compensated trial activities.

The interview guide was developed by the research team with content tailored to whether a participant had quit or was currently smoking. Questions focused on the overall impact of COVID-19 on tobacco use. Interviews were conducted by three different clinical research coordinators who interacted with trial participants during parent trial activities. The interviewers were bachelors level research staff who underwent training in qualitative interviewing methods with didactic teaching and skills-based practice in qualitative methods by two investigators (KS, GK). A semi-structured interview style was utilized so as to provide a guided list of questions and prompts to be explored while allowing for flexibility and participant influence in directing interviews. The guide was piloted with three individual participants after which it was determined that no substantive changes were required, and the interview guide was utilized for the remaining 40 interviews. Our sample size attempted to balance the need to achieve thematic saturation with the practical limitations of the parent trial. Interview transcripts were reviewed iteratively during the qualitative data collection process to assess whether thematic saturation was achieved prior to reaching the practical limitations of our active trial cohort.

### 2.4. COVID Qualitative Analysis

Interviews were audio-recorded and transcribed verbatim, and transcripts were checked for accuracy and completeness and edited where necessary. Data were prepared for analysis using NVivo qualitative software version 12.0 (QSR International Pty. Ltd., 2012, Doncaster, Australia).

A framework approach guided analysis [16]. Data coding and analysis were completed by a team of four female investigators (KS, SJ, JK, GK) with training in qualitative methods and diverse backgrounds. Backgrounds included a psychiatric clinician-researcher (KS), a clinical research coordinator with a B.A. in psychology (SJ), a project manager with an advance practice nursing degree and certified tobacco treatment specialist (JK), and an internal medicine clinician-researcher (GK). Transcripts were reviewed individually by members of the research team for general familiarity and grasp of content [17]. An initial codebook was developed based on the thematic queries from the interview guide and initial content analysis by team members. The initial codebook was tested on three transcripts and modified in order to settle on a finalized codebook [18]. All transcripts were double coded, with weekly meetings held to review coding by all pairs and ensure a high level of intercoder agreement between each pair (kappa > 0.8). Coding was performed at the passage level, meaning that all continuous speech by one speaker is considered a single code until the speaker changes.

Following completion of coding, further analysis entailed querying coding reports and a further round of transcript review, first individually and then discussed in team meetings, to identify content patterns and emergent themes. Cross-case analyses were utilized to compare experiences and outcomes based on quantitative participant characteristics. This included, for example, comparing participants as cases to explore whether patterns of experiences with smoking and stress were the same or different for high resiliency and low resiliency cases. Coding matrices [19] were utilized to characterize emergent themes by participant demographic characteristics and quantitative survey responses. Next, emergent themes and patterns were triangulated with existing health behavior frameworks and theories [10]. The emergent themes were found to map most closely to the Transactional Model of Stress Framework [20,21,22,23], allowing for validation and expansion of this model.

Procedural measures to promote rigor [24] included establishment and training of the coding team, analysis by individuals with different research backgrounds to limit the introduction of personal biases, double coding of all transcripts, ongoing recalibration meetings to ensure the reliability of the coding team and to reduce coding drift, triangulation with established theories, and documentation of all coding and analytic procedures.

## 3. Results

### 3.1. Participant and Call Characteristics

Participants (N = 39) smoked on average ~20 cigarettes per day at study entry. During the baseline survey while hospitalized, moderate-to-severe symptoms of depression (54%) and anxiety (49%) were common, with moderate resilience (mean 3.5 on a scale from 1–5) and relatively high average pessimism (mean 13.4, score range from 0–24 higher scores indicating higher optimism/lower pessimism). See Supplemental Table S1. The mean call duration was 12 min and 7 s (standard deviation [SD] = 4.05 min, range 5.3–22.85). All qualitative interviews were completed between the hours of 0800 and 1700. Fourteen individuals had quit tobacco at the time of their qualitative interview. Of the remaining 25 individuals, 12 reported increased smoking, 7 reported decreased smoking, and 6 reported no change in the amount smoked during COVID-19. A majority of interview participants (n = 31, 79%) reported the perception of increased susceptibility to COVID-19 given their status as a current or former smoker, with 52% (n = 13) of current smokers noting a likely reduction in this risk if they were to quit [25].

### 3.2. Identified Impacts and Reactions to COVID-19

**Figure 1:**
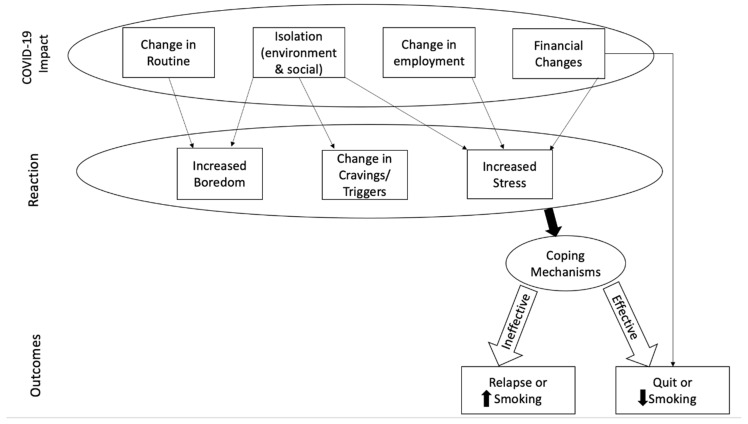
Proposed mechanism of COVID-19 impact on tobacco use.

Four main impacts of the COVID-19 pandemic reached thematic saturation: change in routine, social and environmental isolation, change in employment, and financial changes. Participants’ reactions to these impacts included boredom, change in tobacco cravings and triggers, and increased stress. Whether an individual employed effective or ineffective coping mechanisms to deal with these reactions resulted in either increased tobacco use, decreased use, new tobacco cessation, or sustained cessation during the pandemic. In some cases, financial challenges directly decreased smoking behavior when individuals were not able to continue to purchase cigarettes due to a lack of resources. See Figure 1.

Table 1 highlights identified COVID-19 impacts and reactions, with example quotes from qualitative interviews demonstrating participant experiences and, where applicable, positive and negative effects on tobacco use behavior.

### 3.3. Stress during COVID-19

#### 3.3.1. Coping Mechanisms

Two patterns emerged through qualitative interviews in examining the effectiveness of coping mechanisms. Individuals who increased or continued previous levels of smoking during COVID-19 commonly utilized smoking as their primary coping mechanism, as well as the use of strategies involving exercise and physical components. In contrast, individuals who reduced or quit employed a greater use of companionship and cognitive distractions.

##### Examples

- Increased smoking: smoking as a primary coping mechanism
  ○ “I think people stress. And if you smoke, that’s your go-to. You know what I mean? You’re not thinking about putting on a patch. You’re like, ‘I want a cigarette’… It’s detrimental to people who [are] trying to quit… and people panic. Smokers, that’s the first thing they’re picking up.”—Male, MA
- Continued smoking: physical activity as a primary coping mechanism
  ○ “Well, like I said, the extra thoughts of stress of things going on, of getting it, of my family getting it, has not put me in the best position mentally to work my program… I do my walking and my exercises… to cope with my stress.”—Male, PA
- Decreased smoking: cognitive (prayer) and companionship (pets)
  ○ “Because I put my faith in the good Lord… And that has helped me to keep my stress level down… I do that and pay attention to my little dog and stuff like that. And he’s a good stress breaker, too. Now, as long as I can keep my stress level down, I’m not smoking as much.”—Female, TN
- Remained quit: cognitive strategies and use of companionship
  ○ “My poor family deals with the agitation part of my life. I feel really bad for them but it’s just who I am now, until I get over the urge of wanting to smoke. And I know it’s been seven months, but I won’t lie. I think about it every day… Normally, I take a deep breath and I count to 10 and I do the, ‘It’s going to be okay. I’m going to be okay’. And that calms me down.”—Female, PA

#### 3.3.2. Impact of Baseline Characteristics on Stress

- Low optimism: Twelve of the 39 individuals interviewed scored between 0–13 on the LOT-R which is consistent with low optimism. Among this group, the most common mechanism for dealing with increased stress was smoking. In contrast to those with moderate-to-high optimism, those with low optimism more often referenced boredom as a smoking trigger. Nine of the 12 individuals with low optimism were actively smoking at the time of interview, and five reported increasing smoking during COVID-19.
  ○ “I mean, really, there again, it’s just I know that I’ve increased. When I’m sitting around and not doing anything, that’s when I want to smoke and I just constantly smoke.”—Female, TN
  ○ “My stress went through the roof. And the only way I can cope is smoking.”—Female, PA
- Low resilience: Six individuals interviewed scored a 1–2 on the BRS, classified as low resilience. Smoking as a coping mechanism for stress was also more common for this group compared to individuals with moderate to high resilience, and there was noted an increased reference to increased worry for self and others. All six individuals were actively smoking at the time of interview, five reported increasing smoking during COVID-19.
  ○ “I live in fear now.”—Female, PA
  ○ “It’s stressful. We have some grandchildren under eight and I worry about them. In general, with everything that’s going on. It’s nerve-wracking.”—Male, PA
  ○ “I worry about other people having it and don’t know it, you know?”—Female, PA
- Moderate to high anxiety and depressive symptoms: Nineteen individuals were classified as having moderate to high anxiety symptoms and 21 individuals were classified as having moderate to high depressive symptoms during hospitalization at study entry. In qualitative interview, this group more often mentioned smoking as a primary coping mechanism or mentioned concern regarding the risk of contracting COVID, and reported increased financial stress, as compared to those with lower symptomatology.
  ○ “I’ve tried to quit several times. I know that I need to quit smoking because of my health issues but, however, it’s not that easy. It’s not, especially at a time like this. Somebody could have been stopped smoking, and with this going on they’ll light up. Nerves. Worry. We don’t know what this stuff is. It’s killing people.”—Male, PA
  ○ “It’s something I really don’t want to do anymore but it’s just like there’s so much stress in my house because everybody’s home, nobody’s working. It’s just like it calms me down.”—Female, TN
  ○ “I’m on disability so I only get so much. And by the time I pay my bills, I’m totally dead broke. And when I got to the hospital, I needed a lot of things that my insurance didn’t pay for… And it’s just crazy. So yes, I’m not doing well money-wise.”—Female, MA

### 3.4. Stress during COVID-19

**Figure 2:**
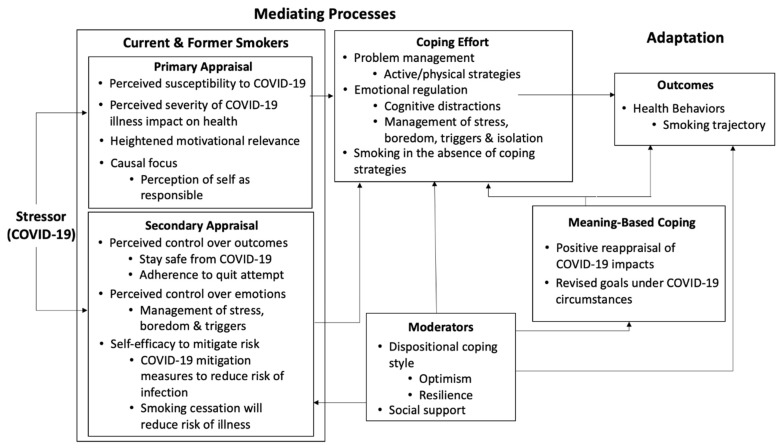
Impact of COVID-19 on a smoking trajectory through mediating processes as outlined by the Transactional Model of Stress. Figure adapted from Glanz et al. [23], based on the models proposed by Lazarus, Cohen, Folkman, and Moscowitz [20,21,22].

The above findings were triangulated with existing health behavior theories [10]. Our findings related to the impact of COVID-19 on stress and tobacco use aligned with the Transactional Model of Stress [20,21,22,23]. As illustrated in Figure 2, identified constructs have been mapped onto the mediating processes of this model in response to the stressor of COVID-19 and ultimately impacting smoking trajectory.

- Primary appraisal as defined by this model refers to an individual’s assessment of a stressor, including their perceived susceptibility to and severity of the threat, taking into account their current motivation and to what extent they believe themselves responsible. Most individuals reported heightened susceptibility and increased likelihood of severe illness and reported heightened motivation to quit. Some specifically mentioned themselves as responsible for their current increased risk.
  ○ Perceived susceptibility and severity: “What changed for me… I’m just afraid that somebody in my family is going to get it and die or me because I have COPD really bad. And I’m supposed to be on oxygen, so my lungs are already really bad for my age. So if I get [COVID-19], the chances are I probably won’t make it out of it.”—Female, PA
  ○ Causal focus: “If I’m constantly smoking and doing everything that I’m not supposed to be doing, I don’t know. I just think that people that are doing that on purpose, not that they deserve to die or anything like that, God forbid, I don’t mean that at all. But it’s like, ‘You weren’t helping yourself.’—Male, MA
- Secondary appraisal as defined by this model refers to an individual’s assessment of perceived control over outcomes and emotions in response to a threat and includes the construct of self-efficacy. Specific to COVID-19, participants noted varying levels of perceived ability to stay safe from contracting disease, adhere to a quit attempt and manage changes in boredom, cravings, triggers, and stress.
- Coping effort as highlighted above, we observed a distinction in the efficacy of coping methods relating to smoking trajectory between individuals who utilized companionship and cognitive distraction as opposed to exercise, physical components, and/or reverting to smoking. Mapping onto the Transactional Model of Stress aligns this with the distinction between problem management (active/physical strategies) and emotional regulation (i.e., cognitive distraction, stress management).
- Moderators refers to the effects of an individual’s dispositional coping style and available supports on the stress response. As highlighted above, we observed differences in coping efforts and outcomes among individuals with differing levels of optimism, resilience, anxiety, and depression on self-report at baseline hospitalization.
- Meaning-based coping refers to an individual’s ability to view a situation in a positive light and/or utilize coping strategies reflecting core values. Some individuals mentioned an ability to view the various impacts of COVID-19 as facilitators of positive health behavior change, i.e., not being able to smoke in the house or around family, financial limitations preventing spending money on cigarettes.

## 4. Discussion

This qualitative study analyzed the impacts of the COVID-19 pandemic on individuals in a post-hospitalization smoking cessation intervention trial, identifying four main factors—routine, social and environmental isolation, change in employment, and financial challenges—resulting in increased boredom, change in cravings and triggers, and, notably, increased stress. Availability of effective coping mechanisms aside from reverting to a default smoking pathway heavily influenced an individual’s subsequent smoking behavior.

Triangulating emergent themes and patterns with the Transactional Model of Stress provided a useful framework for understanding and expanding on the qualitative analysis and validating emergent findings. Most individuals reported increased perceived susceptibility to COVID-19 given their status as a current or former smoker and many mentioned an increased likelihood of severe illness if they were to contract the disease. This pattern fits within the model’s construct of primary appraisal, Smith and Lazarus note that these factors may compound guilt and anxiety related to the stressor if a stressor has high motivational relevance, i.e., relation to an individual’s health in this case, as well as if there is a perception of the self as the cause of this relevance (i.e., in the case of smoking-related illness) [26].

An individual’s assessment of perceived control over outcomes and emotions in response to a stressor, as well as self-efficacy comprise secondary appraisal under this model. Behavior-specific self-efficacy, described in the context of Social Cognitive Theory, has been demonstrated to heavily influence an individual’s ultimate success [27,28]. Consistent with the model, we found that individuals who were confidently able to enumerate effective coping strategies were more likely to quit or remain quit and that those with lower optimism and resilience (dispositional coping style moderators) were more likely to express a lack of control and reliance on smoking as a default response to stress.

Coping efforts as outlined by the Transactional Model of Stress can take the form of problem management and/or emotional regulation. The model predicts that problem-focused coping strategies will be most adaptive for stressors that are changeable, whereas emotion-focused coping is often most adaptive when the stressor is unchangeable [23]. In line with this reasoning, we found that those who employed companionship and cognitive distractions quit or remained quit during COVID-19, whereas those who relied on physical strategies which may be limited or altered during COVID-19 (i.e., exercise) were not as successful. In the absence of alternative coping strategies, many individuals reverted to smoking behavior.

Finally, in consideration of meaning-based coping, some individuals were able to reinterpret otherwise negative events resulting from COVID-19 (i.e., isolation, financial challenges), as positive impacts in line with their goals of cessation. Individuals referenced quitting or reducing smoking if they were not able to go outside to buy cigarettes (environmental restriction) or did not have the financial means to do so. Changes to the environment and social exposure additionally reduced the presence of triggers for some individuals, i.e., not seeing others smoke.

Our work adds to the literature by building on quantitative research in France, Australia, and the Netherlands that has shown associations between stress and tobacco use during COVID-19 [29,30,31,32]. Two quantitative analyses noted increased perceived vulnerability to COVID-19 among those who smoke with varied responses in smoking behavior [25,33]. A mixed-methods study by Rossof-Verbit et al. noted increased stress and lack of access to coping mechanisms resulted in difficulty quitting for individuals enrolled in tobacco cessation trials in the northeastern United States [34]. Another study in France highlighted the association between depressive symptoms, stress and smoking [35]. While heightened stress was nearly universal among our sample, our work highlighted differences between individuals who reported smoking less or quitting versus smoking more including the successful deployment of coping strategies during the pandemic. For many, smoking was the most available and habituated coping mechanism in this time of heightened stress, particularly among individuals with low optimism, low resilience, and heightened depressive and anxiety symptomatology. Low optimism has been associated with continued smoking in other contexts; our work adds to this literature by highlighting the potential for low optimism to affect the relationships between heightened stress and smoking [36].

Our work has limitations. Consistent with qualitative research methods, our sample size is small but comparable to similar work [34]. Our qualitative data collection was embedded within a larger trial cohort and this also created practical limitations on the sample size. However, our sampling process was guided by thematic saturation and consistent with this, all themes reported here were expressed by multiple participants. We acknowledge that the time of day that the interview was conducted may have affected the interview response. However, the themes we queried were longitudinal, spanning the pandemic and not in-the-moment assessments of an individual’s stress.

## 5. Conclusions

This work fills an important gap in the literature aiming to understand the impacts of events such as the COVID-19 pandemic on tobacco and health behaviors. Our findings show tobacco cessation programs and counseling efforts would do well to identify and qualify the impacts of events such as COVID-19 on levels of stress, boredom and increased cravings or triggers with individuals in their care, thus increasing self-awareness. Special focus should be given to strengthening effective coping skills (minimizing the use of tobacco as a default pathway) and increasing perceived control and self-efficacy. Given the COVID-19 imposed limitations of quarantine and social distancing, coping mechanisms targeting emotional regulation as opposed to those employing problem solving and physical activity may be more effective, as they were for individuals in this qualitative study. Inherent personal characteristics including low optimism, low resilience, and noted symptoms of anxiety and depression may negatively impact perceived self-efficacy, stress management, and smoking behaviors, and could be better addressed if anticipated. The hypotheses generated from this work could be tested in a larger, representative sample and used to inform intervention design. In particular, our results indicate that the Transactional Model of Stress could be helpful in conceptualizing the impact of COVID-19 and processes related to stress on health behaviors, and avenues to consider in tobacco cessation intervention design for mitigating the impact of stress.

## Figures and Tables

**Table 1:** COVID-19 impacts and reactions—participant quotes.

COVID-19 Impacts	Experience	Tobacco Behavior
Change in routine	“So, my everything is upside down now. I’m not going to work, the kids aren’t going to school. They’re doing everything online, so we’re all home all the time. So, yeah, basically everything about my schedule and routine is completely different.” - Female, PA	“It just gives me a lot more downtime. It definitely gives me time in my own mind and my own thoughts. So, I mean, just again, stresses that come on, and you start contemplating these other problems and don’t have this, can’t have that, can’t afford this… then you start smoking.” - Male, MA
Isolation—social	“It makes me feel isolated a lot of times. When I do wish I had someone that could come over and spend some time with me but I don’t want them to come over because I don’t want to get exposed because I don’t know what they’re doing outside of my house and stuff. My parents haven’t been able to come over like they used to because they’re in their 70s.” - Female, TN	“I used to always be outside and in public and I’m a people person. I’m a hugger, you know what I mean? That has killed me. That personal human touch. That stresses me out and makes me want to go smoke.” - Female, MA
Isolation—environment	“Staying pretty much at home has bothered me… The way you got to stay in. Nothing’s moving. I’m losing a lot of work over it.” - Male, PA	Quit smoking “I don’t go outside that much, to store to buy cigarettes or anything. So that’s one of the reasons I quit, because I’d rather stay at home than go outside and buy a pack.” - Male, MA Increased smoking “It gets old after a while… I mean, it’s like being shut in and not being able to go anywhere, it’s really tough when you’re just sitting day in day out with nothing to do… I know that I’ve increased. When I’m sitting around and not doing anything, that’s when I want to smoke and I just constantly smoke.” - Female, TN
Employment	“Absolutely nothing at all [I can do] to get any kind of income to keep my business afloat. Nothing. I mean there’s nothing I can do right now. Like everybody else, I’m just sitting in the house waiting for it to pass.” - Female, PA	“Well, prior to all this… I would go to work. And I worked 10 h shifts. I never once smoked at work. I’m not a person to go to lunch and have a cigarette or say I’m going to have a smoke break. I don’t do that, and that was a big part of the reason, I think, that I smoked such a small amount. And now that I’m home, I find it’s almost something to do. I work from home, but on my lunch break I’ll go outside and smoke a cigarette. And I get super mad at myself, and I do it anyway.” - Female, MA
Finances	“Well, [I have] no income because I don’t get to go to work or look for a job because there’s not many jobs open right now and [I don’t] leave the premises because we could infect other people if we leave.” - Male, TN	Increased smoking “I stress about money, and so I smoke more. When I can’t do anything or help my children or anything like that that bothers me. I smoke more.” - Male, MA Decreased smoking “No, I think it changed for the good way… my health and everything... It helps with my health, [given] my financials. I don’t got $10 to spend. I used to spend $10 every two days when I used to smoke. Now, I’m saving those $10 so it changed me in a good way.” - Male, MA Remained quit “But now, with the virus, I need that money to pay bills because my normal way of making money completely got shut down this year. And I feel that now with the coronavirus going through that. Thank God I quit smoking because now that money can go towards bills. - Female, PA
Boredom	“Oh, I mean, we can’t do anything. We can’t go anywhere unless it is doctor-related or store-related to grab something. So I mean, with everything shut down, there’s nothing you can do. You can’t go anywhere. And it’s just so you can only sit home and do so much that you’re just extremely bored.” - Female, TN	“Well, I did really good for a while. I mean, I just need a cigarette because there’s nothing else to do.” - Female, PA
Cravings/Triggers	“The other thing; my boyfriend smokes, and he smokes a lot, so it’s like… because I’m constantly around it, it’s a trigger for me.” - Female, MA	Increased smoking “But it’s like, when I wake up in the morning and I’m sitting on that deck and I’m looking outside and I see nothing, nobody, nothing, I have nobody and nothing [to do]… after having a few cups of coffee… you want to have something to go with it. And what usually goes with caffeine in the morning? A cigarette.” - Female, MA Remained quit “Oh, I think it’s easier if you don’t see people smoking all the time to not smoke yourself.” - Female, PA
Mood/Stress	“I would say distress. I’m out of work, spending home with kids, my wife. My wife’s not working, the homeschooling. There was a long period of time with the way the weather was; you really couldn’t do anything outside, so we were doing project inside, and that gets stale… It’s a lot more stressful. I would say in even thinking and talking about it right now, [it’s] a little more stressful.” - Male, MA	“It’s made me smoke more. It’s been nerve-wracking going through this… So I’ve smoked more, not knowing the end result of what’s going to become of all this.” - Female, PA

## Supplementary Materials

The following are available online at https://www.mdpi.com/article/10.3390/ijerph18105404/s1, Table S1: Baseline sample characteristics (*n* = 39).
