# Supplementary material for: A Qualitative Study of the Impact of COVID-19 on Smoking Behavior for Participants in a Post-Hospitalization Smoking Cessation Trial
Source: Int J Environ Res Public Health. 2021 May 19;18(10):5404. doi: 10.3390/ijerph18105404 (PMC8158767; doi:10.3390/ijerph18105404)
Supplement: Supplementary file 1 [file ijerph-18-05404-s001.zip › ijerph-1183229-supplementary.pdf]

Supplemental Table S1. Baseline sample characteristics (n=39).

	**N (%)**
Age (mean [SD])	52.6 [12.7]
Gender (female)	22 (56.4%)
Race/ethnicity	
White, non-Hispanic	32 (82.1%)
Black or African American, non-Hispanic	3 (7.7%)
Hispanic	4 (10.3%)
Cigarettes/day (mean [SD]	19.7 [13.1]
Live with another smoker	21 (53.9%)
Home smoking policy	
No smoking allowed at home	20 (51.3%)
Smoking allowed in some places at home	2 (5.1%)
Smoking allowed anywhere at home	17 (43.6%)
Importance of quitting[1]	9.36 [1.06]
Confidence in ability to stay quit[2]	6.85 [2.48]
Depression symptoms (PHQ)	
Mean [SD][3]	10.8 [7.4]
PHQ ≥10[5]	21 (53.8%)
Anxiety symptoms (GAD)	
Mean [SD][4]	10.8 [7.5]
GAD ≥10[6]	19 (48.7%)
Resilience (BRS-mean [SD][7)]	3.48 [1.00]
Life orientation (LOTR-mean [SD][8)]	13.42 [4.37]

[1] Participant self-report of importance of quitting smoking when leaving the hospital from 1 (not at all important) to 10 (very important), assessed at baseline in hospital

[2] Participant self-report of confidence from 1 (not at all confident) to 10 (very confident) of in ability to stay quit for six months post-hospitalization, assessed at baseline in hospital

[3] Measured via Patient Health Questionnaire (PHQ-8) scale, assessed at baseline in hospital

[4] Measured via Generalized Anxiety Disorder (GAD-7) scale, assessed at baseline in hospital

[5] Indicative of moderate-severe depressive symptoms

[6] Indicative of moderate-severe anxiety symptoms

[7] Measured via Brief Resilience Scale (BRS), assessed at baseline in hospital

[8] Measured via Life Orientation Test-Revised (LOT-R), assessed at baseline in hospital
